# Two-in-one: multifunctional poloxamer hydrogel accelerates endometrial regeneration and fertility restoration through synergistic regulation of KGF-2 and NO

**DOI:** 10.1093/rb/rbaf062

**Published:** 2025-06-20

**Authors:** Yijia Zhang, Xinji Wang, Qin Gu, Cuitao Lu, Yingzheng Zhao, Xiaokun Li

**Affiliations:** Department of Reproductive Medicine, The First Affiliated Hospital of Wenzhou Medical University, Wenzhou 325000, China; Department of Pharmaceutics, School of Pharmaceutical Sciences, Wenzhou Medical University, Wenzhou 325035, China; Department of Pharmaceutics, School of Pharmaceutical Sciences, Wenzhou Medical University, Wenzhou 325035, China; Department of Pharmaceutics, School of Pharmaceutical Sciences, Wenzhou Medical University, Wenzhou 325035, China; Department of Pharmaceutics, School of Pharmaceutical Sciences, Wenzhou Medical University, Wenzhou 325035, China; Department of Pharmaceutics, School of Pharmaceutical Sciences, Wenzhou Medical University, Wenzhou 325035, China; Department of Pharmaceutics, School of Pharmaceutical Sciences, Wenzhou Medical University, Wenzhou 325035, China

**Keywords:** endometrial injury, keratinocyte growth factor, nitric oxide, poloxamer hydrogel, regenerative repair

## Abstract

A healthy endometrium is crucial for embryo implantation and pregnancy maintenance. Thin endometrium, reduced glands and fibrosis resulting from infection or mechanical injury, are the primary causes of long-term infertility and poor pregnancy outcomes. Unfortunately, these issues have not been resolved by conventional clinical methods. Keratinocyte growth factor-2 (KGF-2) is an epithelial mitogen that regulates proliferation and migration of epithelial cells. Nitric oxide (NO) is involved in maintaining vascular homeostasis and angiogenesis. Poloxamer-407 (P) hydrogel is a promising topical drug delivery system due to its excellent solution-gel transition properties in response to body temperature. In this study, therapeutic NO gas was first prepared into stabilized microbubbles (NO-MBs). Subsequently, KGF-2 and NO-MBs were encapsulated into micelles of P hydrogel to form a multifunctional temperature-sensitive (28.9–31.8°C) hydrogel (KGF-NO-MBs-P hydrogel). This hydrogel not only exhibited suitable apparent viscosity, bio-adhesive and mechanical properties for application *in situ* but also showed sustained release of KGF-2 and NO. *In vivo*, KGF-NO-MBs-P hydrogel effectively restored endometrial morphology, increased the number of glands and endometrial thickness, reversed endometrial fibrosis and improved pregnancy outcomes by synergistic regulation of KGF-2 and NO. Repair of endometrial injury was closely related to promoting neovascularization, inducing endometrial cell proliferation and epithelialization, inhibiting apoptosis and inflammation and balancing collagen subtypes. Therefore, KGF-NO-MBs-P hydrogel may be useful in promoting endometrial regeneration and fertility restoration through *in situ* microinjection. This study represented a convenient, safe and promising method for repair of endometrial injury.

## Graphical abstract

**Figure:**
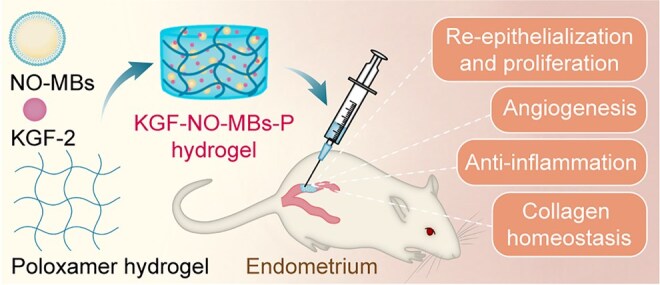

## Introduction

Endometrium is composed of epithelium, stroma and blood vessels, consisting of epithelial cells, stromal cells, endothelial cells, immune cells and stem cells and it is the primary site for embryo implantation and development [1]. Endometrial injury caused by repeated curettage procedures and infection leads to endometrial atrophy, glandular inactivation and poorly vascularized stroma [2]. Severe endometrial damage results in uterine adhesion or endometrial thinning, which leads to abnormal menstruation or amenorrhea, recurrent miscarriages, infertility and pregnancy-related complications [3]. Conventional approaches such as surgery, exogenous hormones (e.g. estrogen and progesterone) and blood flow-enhancing medications (e.g. sildenafil and aspirin) have poor prognosis [4]. These methods have disadvantages such as long treatment cycles and high doses, which increase risks of cardiovascular diseases or tumors [5]. Besides, cell therapy such as colony-stimulating factor, platelet-rich plasma (PRP), stem cells have been used to repair damaged endometrium [6]. However, highly invasive and costly processes of harvesting, low implantation rate and immune rejection, limit application of these treatments [7].

Growth factors secreted by stem cells have received attention. Keratinocyte growth factor-2 (KGF-2), also known as fibroblast growth factor-10 (FGF-10), is a mitogen for epithelial cells. KGF family plays important roles in regulating epithelial cell migration, proliferation and epithelial-mesenchymal interactions [8]. KGF has been shown to promote regeneration of injured skin, oral mucosa and corneal wounds [9]. Endogenous KGF expression has been detected in endometrium of mice, rats, rhesus monkeys and humans. Endometrial KGF levels were elevated in mice and rhesus monkeys treated with progesterone, and in women during luteal phase of menstrual cycle. Besides, exogenous KGF was found to inhibit glandular apoptosis during luteal-follicular transition [10]. However, KGF faces great challenges in clinical application due to its short half-life, poor stability and low permeability.

Angiogenesis has been recognized as a critical component for epithelial reorganization [11]. Nitric oxide (NO) is an important second messenger involved in maintenance of vascular homeostasis and angiogenesis [12]. NO effectively regulates vasodilatory tone, platelet aggregation and adhesion, inflammatory and immune responses, and apoptosis [13]. NO has been shown to accelerate healing of acute or chronic wounds by modulating angiogenesis and inflammation [14]. Additionally, NO promoted granulation tissue formation and scarless healing by modulating homeostasis of extracellular matrix (ECM) [15]. NO microbubble (NO-MB) is an emerging NO delivery strategy, in which the gas is surrounded by a layer of biocompatible biopolymers, surfactants and lipids. In previous studies, NO-MBs were successfully administered intravenously for treatment of thrombosis and ischemia-reperfusion injury [16]. However, the localized use of NO-MBs in clinical environment is challenging due to their short lifespan in wound areas.

Endometrial regeneration is a long-term process that requires a novel biocompatible scaffold to support sustained release of KGF-2 and NO-MBs to prolong bioactivity. Hydrogel is a commonly used biomaterial for tissue repair, providing a scaffold compatible with cell proliferation and regeneration [17]. Therapeutic factors can be loaded into the hydrogel and released sustainably, maintaining high localized concentrations and reducing administration [18]. Poloxamer is a poly (ethylene oxide)-poly (propylene oxide)-poly (ethylene oxide) copolymer (PEO-PPO-PEO), and is widely used *in situ* as an hydrogel-forming material due to its temperature-sensitive solution-gel transition properties [19]. It has low-toxicity, biocompatibility and biodegradability [20]. Poloxamer hydrogel containing a-FGF showed significant therapeutic efficacy in wound treatment [21]. Furthermore, 17β-estradiol-loaded poloxamer hydrogel promoted regeneration of damaged endometrium [22]. Therefore, poloxamer (P) hydrogel may be an effective drug delivery system for KGF-2 and NO-MBs in endometrial injury.

**Figure 1.**
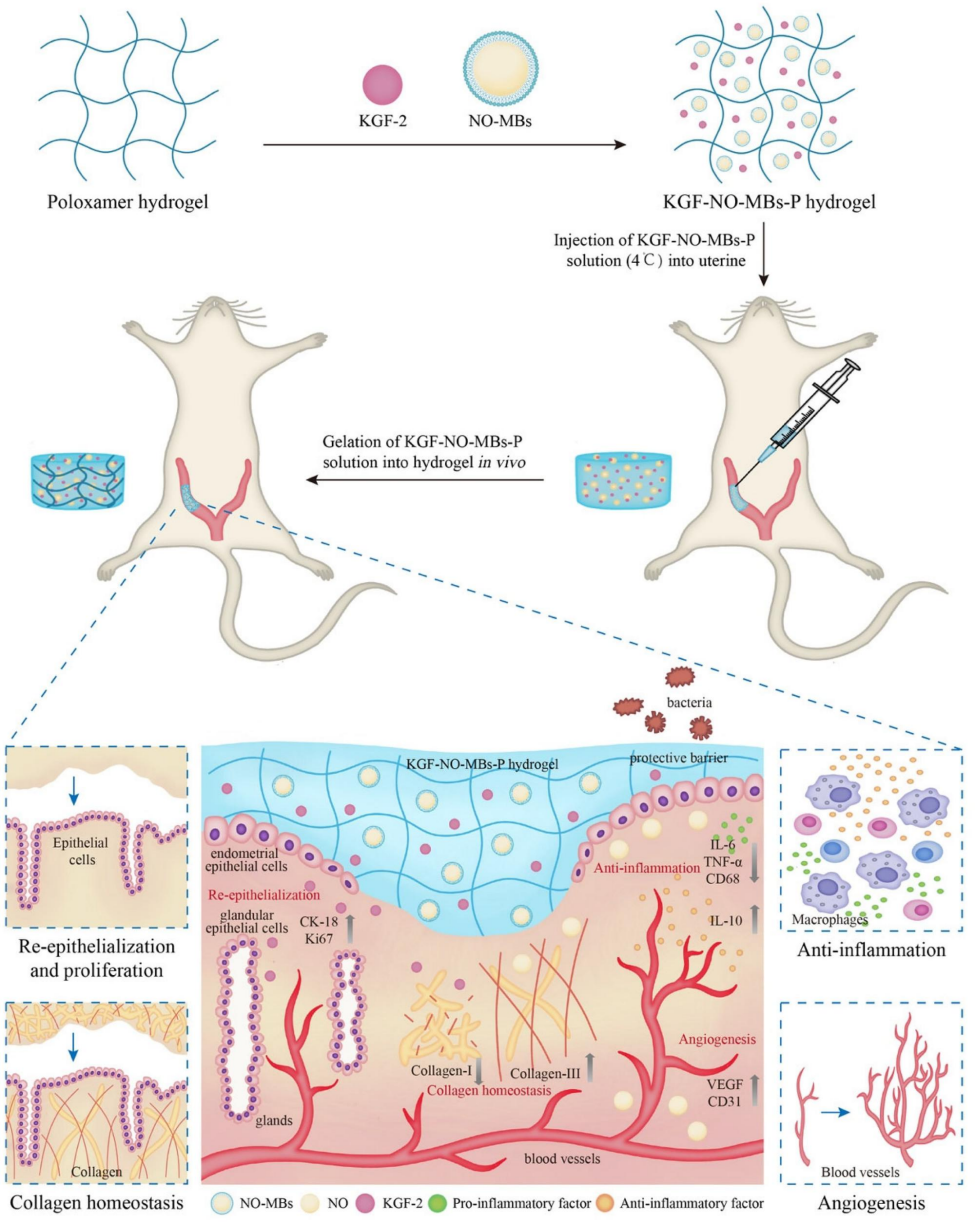
Schematic illustration of applying KGF-NO-MBs-P hydrogel for regeneration of injured endometrium.

In this study, we explored whether co-delivery of KGF-2 and NO can enhance endometrial regeneration and fertility restoration. KGF-NO-MBs-P hydrogel was prepared, and impacts of KGF-2 and NO-MBs on temperature sensitivity, rheological properties, bio-adhesion and release behaviors of P hydrogel were investigated. Therapeutic effects and potential mechanisms of KGF-NO-MBs-P hydrogel on morphological reconstruction, functional regeneration and fertility recovery of injured endometrium were evaluated through cellular assays *in vitro* and animal experiments *in vivo* (Figure 1). KGF-NO-MBs-P hydrogel may offer a new strategy for treating intractable damaged endometrium.

## Materials and methods

### Reagents and antibodies

Pluronic^®^F-127 (P407, ploxamer-407) and Kolliphor^®^P-188 were purchased from Sigma-Aldrich (Missouri, USA). Hydrogenated soybean phospholipids (HSPC) and 1,2-octadecanoyl-sn-glycero-3-phosphocholine (DSPC) were obtained from Avite Pharmaceutical Technology Co., Ltd. (Shanghai, China). Tert-butanol was purchased from Xilong Scientific Co., Ltd. (Guangzhou, China). Tween-80 and fluorescein isothiocyanate (FITC) were acquired from Solarbio Scientific Co., Ltd. (Beijing, China). KGF-2 was provided by Wenzhou Medical University (Wenzhou, China). NO Assay Kit was purchased from Beyotime (Shanghai, China). Human umbilical vein endothelial cells (HUVECs) were supplied by Shanghai Zhongqiaoxinzhou Biotechnology Co., Ltd. (Shanghai, China). Dulbecco’s modified Eagle's medium (DMEM) and fetal bovine serum (FBS) were bought from Invitrogen (California, USA). Hematoxylin and Eosin (HE) Staining Kit and Masson’s Trichrome Staining Kit were obtained from Beyotime (Shanghai, China). Antibodies against Ki67, CD31, CD68, Collagen-I and Collagen-III were purchased from Abcam (Cambridge, UK). Antibodies against VEGF, Caspase-3, IL-6, IL-10, CK-18 and TNF-ɑ were purchased from Proteintech (Rosemount, USA).

### Preparation of NO-MBs and KGF-NO-MBs-P hydrogel

#### NO-MBs

**Figure 2.**
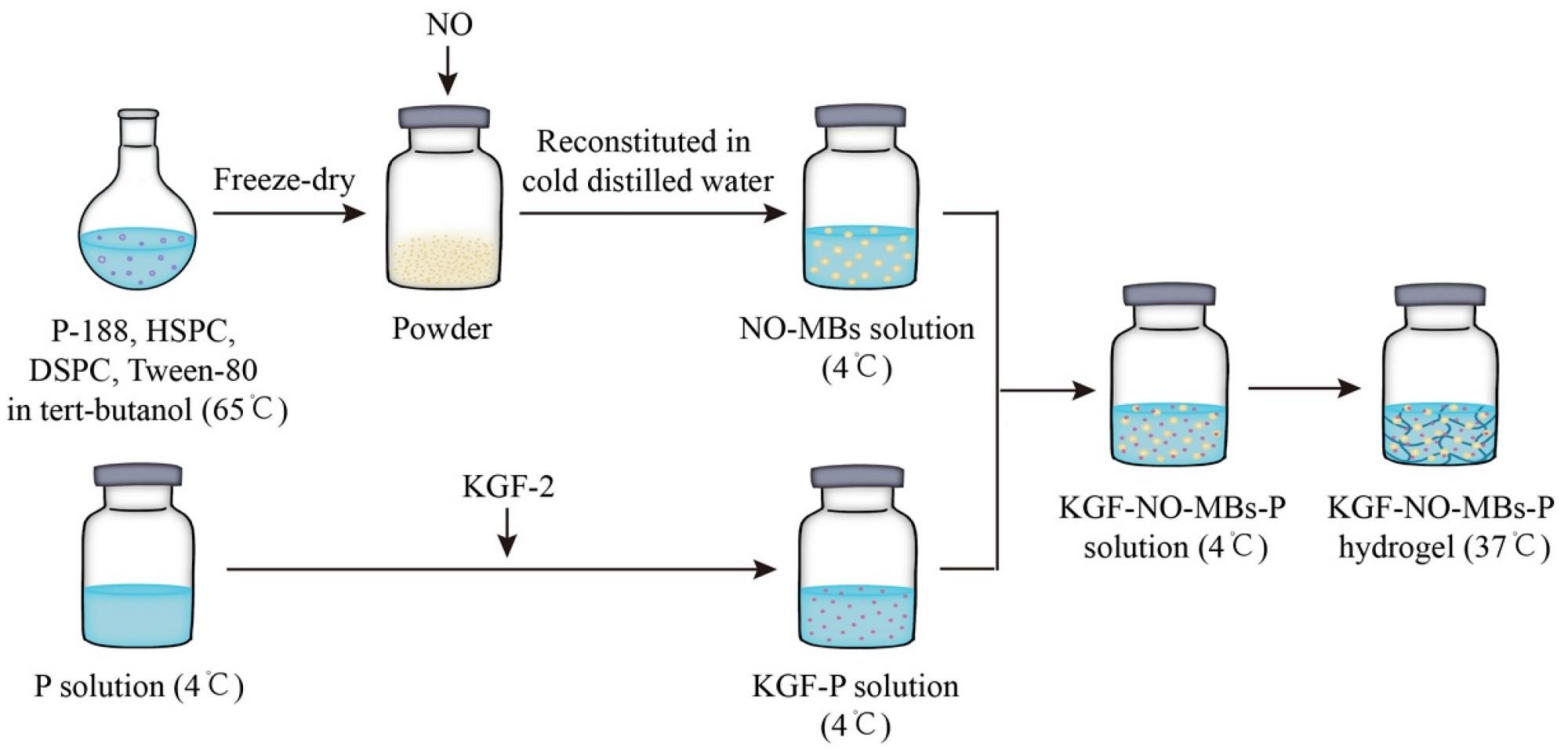
Preparation of KGF-NO-MBs-P hydrogel.

NO-MBs were prepared by lyophilization method. P-188 (200 mg), DSPC (4 mg), HSPC (2 mg) and Tween-80 (20 mg) were dissolved in tert-butanol (4 mL) at 65°C. Dissolved lipid solution was lyophilized in a Freezone freeze dryer (Labconco, USA) at the pressure of 5 × 10^−4 ^Pa for 24 h. A certain amount of dried powder was placed into a sealed glass bottle. First, the bottle was filled with N_2_ through a three-way valve to replace the air. Then, NO was injected into the bottle from the bottom. NO-saturated powder was dissolved in distilled water to form NO-MBs solution (4°C). Further, P-407 powder was dissolved in distilled water to form P hydrogel (4°C) [23]. NO-MBs solution was then mixed with cold P hydrogel solution to produce NO-MBs-P hydrogel (Figure 2).

#### KGF-NO-MBs-P hydrogel

To prepare KGF-NO-MBs-P hydrogel, a concentrated stock solution of KGF-2 was first prepared by dissolving KGF-2 lyophilized powder in distilled water (4°C). Then, it was mixed in cold P hydrogel to form KGF-P hydrogel. Subsequently, KGF-NO-MBs-P hydrogel was formed by adding NO-MBs solution. Formulations with different concentrations of NO-MBs were prepared based on the fixed KGF-2 concentration of 2.5 mg/mL and P hydrogel (17%) [24] (Figure 2).

### Physicochemical characterization of KGF-NO-MBs-P hydrogel

#### Morphology of NO-MBs

Two-dimensional (2D) morphology images of NO-MBs in NO-MBs solution and NO-MBs-P hydrogel were observed using inverted phase-contrast microscope (Leica Microsystems Inc., Wetzlar, Germany).

#### SEM of NO-MBs-P hydrogel

To observe micromorphology, hydrogels were frozen at −80°C, and then, lyophilized. Samples were placed on a copper sheet with conductive tape, cross-sectioned and sputter-plated with gold using an automatic sputter-coater. Subsequently, they were observed using a scanning electron microscope (SEM, Hitachi, H-7500, Japan).

#### Apparent viscosity and gelation temperature of KGF-NO-MBs-P hydrogel

Apparent viscosity and gelation temperatures of hydrogels were measured using a coaxial cylindrical rheometer (NDJ-8 S, Shanghai Pingsuan Co. Ltd., Shanghai, China). Apparent viscosity was measured within a temperature range from 15 to 45°C, and temperature–viscosity curves were obtained. Gelation temperatures were represented by the point at the maximum slope of temperature–viscosity curve. Each test was performed three times.

#### Rheological characterization of KGF-NO-MBs-P hydrogel

Rheological behaviors of hydrogels were analyzed using a hybrid rheometer (Discovery HR-2, TA Corporation, USA). Samples were placed under a conical plate with a gap distance of 25 μm. First, temperature-sweeps were performed at the frequency of 10 rad/s and strain of 1%, with temperature ranging from 10 to 45°C, followed by 5-min time-sweeps at 37°C. Then, frequency-sweeps were performed at the strain of 1% and 37°C, with frequency increasing from 1 rad/s to 150 rad/s. Finally, strain-sweeps were performed at the frequency of 10 rad/s and 37°C, with strain ranging from 0.1% to 120%. Each test was repeated three times.

#### Adhesion strength of KGF-NO-MBs-P hydrogel

Lap-shear test was used to assess biological adhesion. Briefly, a 20 × 20 mm rat skin was used to simulate biological tissue surface and pasted on a glass sheet. Next, 200 µl of each hydrogel was applied uniformly on biological tissue surface. Then, two glass sheets were overlapped and bonded on Instron machine. Subsequently, it was loaded to fracture with a strain rate of 5 mm/min at 37°C. The maximum force was measured versus displacement to characterize adhesion. Each test was conducted three times.

#### In vivo intrauterine retention of KGF-NO-MBs-P hydrogel

To test *in vivo* retention of KGF-NO-MBs-P hydrogel, KGF-2 was first labeled with FITC. Then FITC-KGF-NO-MBs solution and FITC-KGF-NO-MBs-P hydrogel were prepared and injected into right uterine cavity of rats respectively (*n* = 15/group). Three rats of each group were euthanized at each predetermined time point (2, 8 h and 1, 3, 7 days) after injection. Then, uteri were collected and rinsed with saline. Fluorescence imaging of uteri was performed using *in vivo* IVIS spectral imaging system.

### *In vitro* release of drugs from KGF-NO-MBs-P hydrogel

#### NO-MBs

Release profiles of NO were investigated in PBS at 37°C. Briefly, 1 mL of NO-MBs solution, NO-MBs-P hydrogel or KGF-NO-MBs-P hydrogel was loaded into dialysis membranes (3500 Da) and incubated with PBS as release medium in air bath at 37°C, with continuous shaking at 120 revolutions per minute. At each time point (0, 0.5, 1, 2, 3, 4, 5, 6 h), 50 µl of sample was taken and replaced by an equal volume of fresh medium, then, detected by NO Assay Kit. Experiments were performed three times.

#### KGF-2

Release behaviors of KGF-2 were investigated at 37°C in PBS. Briefly, 1 mL of KGF-2 solution, KGF-P hydrogel or KGF-NO-MBs-P hydrogel was placed in dialysis bags (100 kDa), dialyzed against PBS at 37°C and shaken at 120 revolutions per minute in air bath. At each preset time point (0, 12 h and 1, 2, 3, 4, 5, 6, 7 days), 50 µl of sample was taken and an equal volume of fresh medium was replenished. Then, samples were analyzed using Bradford Protein Assay Kit for KGF-2. Experiments were repeated three times.

### Cellular experiments

#### Cell culture

HUVECs were cultured in high-glucose Dulbecco's Modified Eagle's Medium (DMEM) containing 10% fetal bovine serum (FBS) and 100 U/mL penicillin-streptomycin. Cells were cultured in a 95% humidity incubator (Thermo, San Diego, CA, USA) containing 5% carbon dioxide at 37°C.

#### Cell viability in response to KGF-NO-MBs-P hydrogel

HUVECs were cultured in 96-well plates at a density of 5 × 10^3^ cells/well for 24 h at 37°C. Then, cells were incubated with different concentrations of P-407, KGF-2 or NO-MBs for 12 h. On this basis, cells were also incubated with P hydrogel, KGF-P hydrogel or KGF-NO-MBs-P hydrogel for 12 h. Additionally, cells were pretreated with 200 μmol/l H_2_O_2_ (diluted from 3% H_2_O_2_ by DMEM) for 2 h to simulate injury [23], followed by incubation with P hydrogel, KGF-P hydrogel or KGF-NO-MBs-P hydrogel for 12 h. After these steps, cells were incubated with Cell Counting Kit-8 (Beyotime, Shanghai, China) for 1 h. The optical density (OD) at 450 nm was measured using a microplate reader (SpectraMax190, USA). Experiments were conducted three times.

#### Cell live/dead assay

HUVECs were cultured in 12-well plates at a density of 2 × 10^4^ cells/well. Then cells were pretreated with H_2_O_2_ for 2 h and incubated with P hydrogel, KGF-P hydrogel or KGF-NO-MBs-P hydrogel for 12 h. Subsequently, cells were incubated with Calcein/PI Live/Dead Viability/Cytotoxicity Assay Kit (Beyotime, Shanghai, China) for 30 min, and examined under a fluorescence microscope (ECLIPSE TI-S, Nikon, Japan). Results from three randomized regions were analyzed using ImageJ software (National Institutes of Health, Bethesda, Maryland, USA).

#### Cell ROS assay

HUVECs were cultured in 12-well plates at a density of 2 × 10^4^ cells/well. Cells were pretreated with H_2_O_2_ for 2 h, and then, incubated for 12 h with P hydrogel, KGF-P hydrogel or KGF-NO-MBs-P hydrogel. Reactive oxygen species (ROS) was assessed using ROS Assay Kit (Beyotime, Shanghai, China) and observed by fluorescence microscopy to evaluate antioxidant effects. Results of three random fields of view were analyzed using ImageJ software.

#### Tube formation assay

HUVECs were cultured at a density of 3 × 10^4^ cells/well in 96-well plates pre-coated with ABW^®^ Matrigel gel (ABWbio, Shanghai, China). After adhesion, cells were incubated with P hydrogel, KGF-P hydrogel or KGF-NO-MBs-P hydrogel for 4 h. Changes of tube morphology in three random fields of view were assessed by inverted phase-contrast microscope and analyzed by angiogenesis analyzer of ImageJ software.

#### Cell migration assay

HUVECs were incubated at a density of 2 × 10^5^ cells/well in 6-well plates for 12 h to form a confluent monolayer. Then, the monolayer was scratched with a pipette tip to form a straight line. Subsequently, cells were incubated with P hydrogel, KGF-P hydrogel or KGF-NO-MBs-P hydrogel for 48 h. Images of scratched area were recorded from three random fields at predetermined times (0, 24, 48 h) using an inverted phase-contrast microscope. In addition, cells were also added to the upper chamber of a transwell analyzer filter and incubated with P hydrogel, KGF-P hydrogel or KGF-NO-MBs-P hydrogel for 12 h, while complete medium was added to the lower chamber. Cells in the upper chamber membrane were fixed and stained with 0.5% crystalline violet, then quantified by an inverted phase-contrast microscope. After the above steps, scratched area healing rates and numbers of migrated cells from three randomized fields were calculated using ImageJ software.

### Animal experiments

#### Rat model of endometrial damage

This study was approved by the Animal Care and Use Committee of Wenzhou Medical University (Ethics No. wydw2023-0088) and was performed in accordance with the National Institutes of Health Guide for the Laboratory Animals. Eight-week-old female Sprague-Dawley rats (230–250 g) (SPF Biotechnology Co. Ltd, Beijing, China) were housed in standard cages with an ambient temperature of 22 ± 1°C, a relative humidity of 50 ± 1%, a light/dark cycle of 12 h/12 h and unlimited access to food and water. Vaginal smears were obtained every morning to select rats with regular estrous cycles for experiments, as surgery was performed at diestrus stage. Endometrial injury was modeled by intrauterine injection of anhydrous ethanol [25]. Briefly, rats were anesthetized by intraperitoneal injection of 2% sodium pentobarbital (0.3 mL/100 g) and uterus was exposed through a vertical incision in the midline of abdomen. The proximal and distal ends of right uterus were clamped with two vascular forceps, then 5 mL anhydrous ethanol was injected into uterine cavity, and allowed to flow out through a thin syringe needle. The damaged segment was then rinsed with saline and gently squeezed to remove ethanol, followed by injection of 100 μl cold saline, P hydrogel, KGF-P hydrogel or KGF-NO-MBs-P hydrogel. Rats in Normal Group were injected intrauterine with only saline instead of anhydrous ethanol. The uterus was later placed back into abdominal cavity, and then, the abdominal muscles and skin were sutured. All animals received penicillin intramuscularly three times afterwards.

#### Groups design and experimental treatments

Rats were randomly divided into five groups (*n* = 12/group):

- Normal Group: injected 5 mL physiological saline;
- Control Group: injected 5 mL anhydrous ethanol, followed by 100 μl physiological saline;
- P Group: injected 100 μl P hydrogel after ethanol-induced injury;
- KGF-P Group: injected 100 μl KGF-P hydrogel after ethanol-induced injury;
- KGF-NO-MBs-P Group: injected 100 μl KGF-NO-MBs-P hydrogel after ethanol-induced injury.

Seven days after injection, 6 rats in each group were euthanized, then uteri were excised for subsequent analysis. The remaining 6 rats in each group were used for fertility experiments.

#### Histological analysis

Collected uterine tissues were fixed overnight with 4% paraformaldehyde. Then, they were dehydrated with graded ethanol, embedded in paraffin and cut into 5-μm thick sections. Sections were then stained with H&E Staining Kit and Masson Staining Kit. Under an inverted phase-contrast microscope, the mean endometrial thickness was measured in four orientations (0°, 90°, 180° and 270°) of five random H&E-stained images, and the number of glands was counted. Additionally, five Masson-stained sections were randomly selected to quantify the extent of endometrial fibrosis using ImageJ software.

#### Immunohistochemical and immunofluorescent staining

For immunohistochemistry:

- Paraffin sections of uterine tissues were deparaffinized and rehydrated. Then, they were incubated in 3% H_2_O_2_ for 15 min at 37°C.
- Sections were incubated in heated sodium citrate (Sinopharm Chemical Reagent) for antigen repair.
- After that, they were blocked with 5% bovine serum albumin (BSA) (Beyotime) for 45 min at 37°C and then incubated with primary antibodies overnight at 4°C. The primary antibodies included anti-Caspase-3 (1: 200, 66470-2-Ig, Proteintech), anti-IL-6 (1: 200, 66146-1-Ig, Proteintech), anti-TNF-α (1: 200, 17590-1-AP, Proteintech) and anti-IL-10 (1: 200, 60269-1-Ig, Proteintech).
- Sections were washed with PBS and incubated with secondary antibodies for 1 h at 37°C. The secondary antibodies included Goat anti-rabbit recombinant secondary antibody (H+L) (ready to use, Cat No. RGAR011, proteintech) and Goat anti-mouse recombinant secondary antibody (H+L) (ready to use, Cat No. RGAM011, proteintech).
- Then, they were exposed to a DAB Chromogen Kit (ZSGB-BIO, Beijing, China), stained with hematoxylin and covered with glass coverslips.
- Three randomly selected areas were imaged using an inverted phase-contrast microscope and analyzed using ImageJ software.

  For immunofluorescence staining:

- Uterine tissues were embedded in optimal cutting temperature (OCT) compounds, rapidly frozen and cut into 5-μm sections.
- Sections were permeabilized with 0.1% Triton X-100, blocked with 5% BSA and incubated with primary antibodies at 4°C overnight. The primary antibodies included anti-VEGF (1: 200, 26157-1-AP, Proteintech), anti-CD31 (1: 500, ab182981, Abcam), anti-CK-18 (1: 200, 10830-1-AP, Proteintech), anti-Ki67 (1: 200, ab16667, Abcam), anti-CD68 (1: 200, ab201340, Abcam), anti-Collagen-I (1: 200, ab34710, Abcam) and anti-Collagen-III (1: 200, ab184993, Abcam).
- Sections were washed with PBS and incubated with secondary antibodies for 1 h at 37°C. The secondary antibodies included Goat anti-rabbit IgG (H+L) FITC (1: 200, BS10950, Bioworld) and Goat anti-mouse IgG (H+L) Rhodamine (TRITC) (1: 200, BS11502, Bioworld).
- Finally, sections were mounted with 4′, 6-diamidino-2-phenylindole (DAPI) and visualized using a fluorescence microscopy.
- Three randomly selected fields of view were semi-quantified using ImageJ software.

#### Fertility testing

Seven days after surgery and injection, six rats in each group were mated with male Sprague-Dawley rats at a ratio of 2:1. The day when a vaginal plug present was considered as day 0 of pregnancy. Rats were euthanized at mid-late gestation (day 17–19) and the number of embryos implanted in uteri was examined.

### Statistical analysis

Data were shown as Mean ± SD and analyzed using Graphpad Prism 9 software (GraphPad Software Inc., San Diego, California, USA). Student's *t*-test was performed to determine significance of differences between pairs. For significant differences between multiple groups, one-way analysis of variance (ANOVA) was used with Tukey’s test. Results of pregnancy rate were compared by Chi-square (or Fisher's exact) tests. *P* < 0.05 was considered statistically significant.

## Results

### Characterization and stability of NO microbubbles

**Figure 3.**
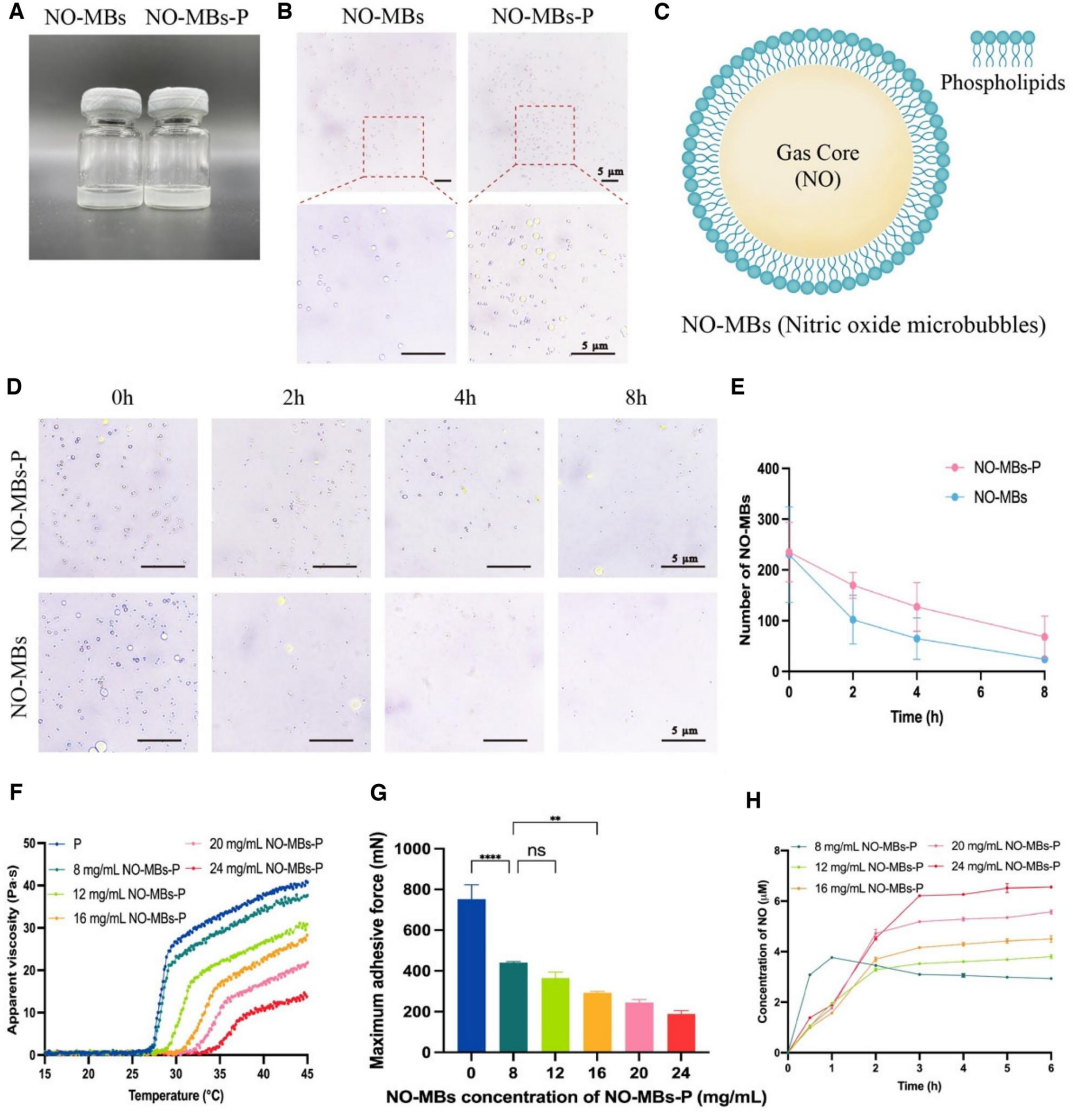
Characterization of NO-MBs and NO-MBs-P hydrogel. (**A**) Macroscopic appearance of cold NO-MBs solution and NO-MBs-P hydrogel. (**B**) NO-MBs in NO-MBs solution and NO-MBs-P hydrogel under optical microscopy. (**C)** Diagram of NO-MBs. (**D** and **E**) Microscopy images and numbers of NO-MBs in NO-MBs solution and NO-MBs-P hydrogel. (**F**) Apparent viscosity–temperature curves of NO-MBs-P hydrogel. (**G**) Maximum adhesive force of NO-MBs-P hydrogel. (**H**) NO release curves of NO-MBs-P hydrogel. *P < 0.05, **P < 0.01, ***P < 0.001, ****P < 0.0001, ns, not significant.

NO-MBs solution and cold NO-MBs-P hydrogel both appeared as homogeneous curd-like suspensions to the naked eye (Figure 3A). Under optical microscopy, NO-MBs were homogeneously polydisperse spherical without aggregation (Figure 3B), in which the NO gas is surrounded by a layer of phospholipids (Figure 3C). For stability evaluation, the numbers of NO-MBs decreased with time in both cases. However, the decrease was significantly faster in NO-MBs solution than in NO-MBs-P hydrogel (Figure 3D and E).

### Characterization of NO-MBs-P hydrogel

#### Apparent viscosity and gelling temperature

To optimize concentration of NO-MBs (8–24 mg/mL), gelation properties of NO-MBs-P hydrogel were evaluated (Figure 3F). Compared to P hydrogel, gelling temperature of NO-MBs-P hydrogel increased while apparent viscosity decreased. As concentration of NO-MBs increased from 8 to 24 mg/mL, gelling temperature range increased from 27.3–29.0°C to 35.0–38.4°C, and apparent viscosity of the platform period decreased from 19.9 × 10^3^ mPa·s to 9.6 × 10^3^ mPa·s. For 12 mg/mL NO-MBs, gelling temperature (29.2–31.9°C) of NO-MBs-P hydrogel was consistent with the measured intraoperative abdominal temperature of rats (about 31°C).

#### Adhesion evaluation

In vitro adhesion of NO-MBs-P hydrogel with different NO-MBs concentration (8–24 mg/mL) was examined. As shown in Figure 3G, NO-MBs-P hydrogel produced weaker adhesion than P hydrogel. When concentration of NO-MBs increased from 8 to 24 mg/mL, the maximum adhesive force of NO-MBs-P hydrogel decreased from 440.0 ± 6.1 mN to 188.7 ± 16.0 mN. Although NO-MBs-P hydrogel with 12 mg/mL NO-MBs had weaker adhesion than that with 8 mg/mL, no significant difference was found.

#### In vitro release of NO from NO-MBs-P hydrogel

Release curves of NO from NO-MBs-P hydrogel with different NO-MBs contents (8–24 mg/mL) are shown in Figure 3H. When concentration of NO-MBs was 8 mg/mL, NO showed rapid release in the first hour, with NO concentration peaking quickly, followed by a promptly slowed release and continuously decreased NO concentration. However, NO-MBs-P hydrogel with 12–24 mg/mL NO-MBs showed sustained NO release within 3 h, with NO concentration increasing steadily, followed by a gradually slowed release rate and a stable plateau concentration. Considering gelling properties, adhesion and NO release behavior, 12 mg/mL NO-MBs was selected for preparation hydrogels and following experiments.

### Characterization of KGF-NO-MBs-P hydrogel

#### Macroscopic appearance and micromorphology

**Figure 4.**
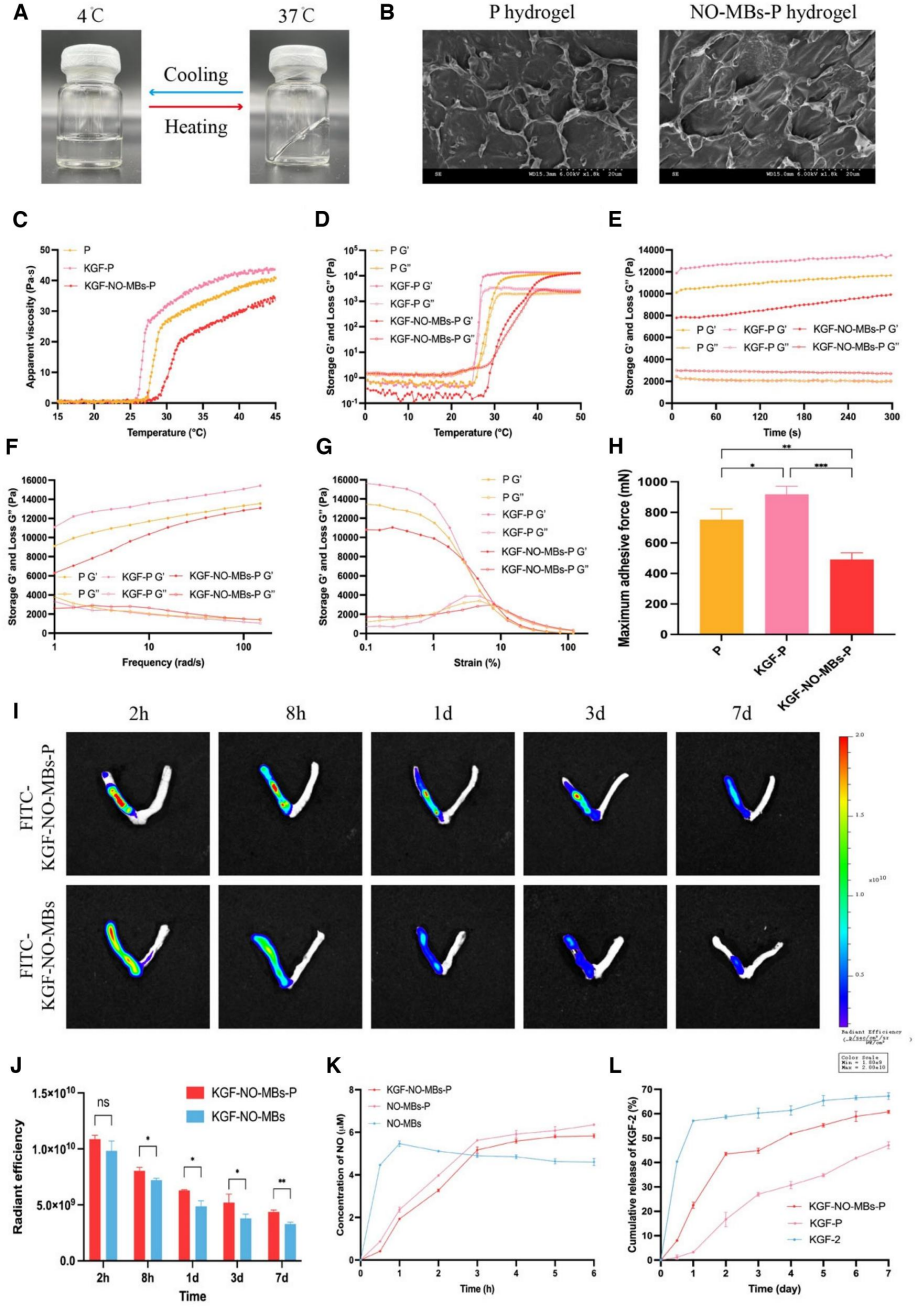
Characterization of KGF-NO-MBs-P hydrogel. (**A**) Macroscopic appearance of P hydrogel. (**B**) SEM images of P hydrogel and NO-MBs-P hydrogel. (**C**) Apparent viscosity–temperature curves of P hydrogel, KGF-P hydrogel and KGF-NO-MBs-P hydrogel. (**D–G**) G′ and G″ of temperature sweep, time sweep, frequency sweep and strain sweep of P hydrogel, KGF-P hydrogel and KGF-NO-MBs-P hydrogel. (**H**) Maximum adhesive force of P hydrogel, KGF-P hydrogel and KGF-NO-MBs-P hydrogel. (**I** and **J**) Fluorescence images and intensity of FITC-KGF-NO-MBs solution and FITC-KGF-NO-MBs-P hydrogel in uterus. **(K**) NO release curves of NO-MBs solution, NO-MBs-P hydrogel and KGF-NO-MBs-P hydrogel. (**L**) KGF-2 release curves of KGF-2 solution, KGF-P hydrogel and KGF-NO-MBs-P hydrogel. *P < 0.05, **P < 0.01, ***P < 0.001, ****P < 0.0001, ns, not significant.

As shown in Figure 4A, P solution could form the clear thermo-reversible hydrogel at 37°C, while remains in the state of micellar solution at 4°C. From the observation of scanning electron microscopy (SEM), P hydrogel showed the microscopic pore structure similar with that of a porous sponge, with interconnected internal pores and uniform pore size. Most pores of NO-MBs-P hydrogel were occupied by microbubbles. This hydrogel exhibited a denser matrix without collapse of microscopic pore structure and change of pore size distribution (Figure 4B).

#### Gelling properties and apparent viscosity

Gelation properties of P hydrogel (17%) containing KGF-2 (2.5 mg/mL) and NO-MBs (12 mg/mL) were evaluated (Figure 4C). The addition of KGF-2 decreased gelation temperature range of P hydrogel from 27.5–29.6°C to 26.1–27.6°C, while apparent viscosity at plateau stage increased from 25.8 × 10^3^ mPa·s to 26.7 × 10^3^ mPa·s. Further, gelling temperature range of KGF-NO-MBs-P hydrogel was 28.9–31.8°C, which was higher than that of P hydrogel and KGF-P hydrogel, and was more consistent with intraoperative abdominal temperature of rats (about 31°C). Although apparent viscosity of KGF-NO-MBs-P hydrogel was lower (19.7 × 10^3^ mPa·s), it was suitable for intrauterine application *in situ* because the reported suitable apparent viscosity is approximately 10 000 mPa•s [26].

#### Rheological characteristic

Rheology tests were carried out. Storage modulus (G′) reflected elastic response and loss modulus (G″) reflected viscous response. Gelation temperature of KGF-NO-MBs-P hydrogel (intersection of G′ and G″) was consistent with viscosity–temperature curve and was closer to intraoperative abdominal temperature of rats (Figure 4D). After gelation, G′ and G″ of KGF-NO-MBs-P hydrogel were independent of time at 37°C (Figure 4E), and G' was higher than G″ throughout frequency change, both demonstrating good stability (Figure 4F). In addition, although G' of KGF-NO-MBs-P hydrogel was lower than P hydrogel and KGF-P hydrogel, strain at the limit of linear viscoelastic region (LVR, about 10%) was comparable among all hydrogels (Figure 4G), indicating favorable ability of KGF-NO-MBs-P hydrogel to endure external strain.

#### Adhesion evaluation

In vitro adhesion of the hydrogel is shown in Figure 4H. The maximum adhesive force of KGF-P hydrogel was stronger than that of P hydrogel (*P* < 0.05), indicating that KGF-2 enhanced adhesion of P hydrogel. However, the addition of NO-MBs weakened adhesion of KGF-NO-MBs-P hydrogel (*P* < 0.001). Therefore, *in situ* retention and release were evaluated subsequently to confirm whether KGF-NO-MBs-P hydrogel is suitable for intrauterine application.

#### In vivo retention and distribution

*In vivo* retention and distribution of KGF-NO-MBs-P hydrogel were investigated by *ex vivo* fluorescence imaging (Figure 4I and J). Fluorescence of right uteri remained confined to the specific region after 2 h of FITC-KGF-NO-MBs-P hydrogel injection, while FITC-KGF-NO-MBs solution rapidly spread along right uteri horn and even reached left uteri. Fluorescence intensity in both groups decreased with time, but the decreasing rate of FITC-KGF-NO-MBs-P hydrogel Group was slower. On the seventh day, FITC-KGF-NO-MBs-P hydrogel showed stronger residual fluorescence in right uteri than FITC-KGF-NO-MBs solution (*P* < 0.01), demonstrating prolonged retention of KGF-NO-MBs-P hydrogel.

#### In vitro release of drugs from KGF-NO-MBs-P hydrogel

NO: NO in NO-MBs solution was rapidly released within 1 h, reaching the maximum content. Then, release rate slowed down along with a decreased content. However, release of NO from NO-MBs-P hydrogel or KGF-NO-MBs-P hydrogel was slower than that from NO-MBs solution. In a later stage, there was sustained release to reach higher maximum contents (Figure 4K).

KGF-2: A burst release of KGF-2 from KGF-2 solution was observed within 1 day, followed by a slowing down of release rate. Compared with KGF-2 solution, release rate of KGF-2 from KGF-P hydrogel was slower, showing a sustained release profile within 7 days. Surprisingly, KGF-NO-MBs-P hydrogel exhibited a faster KGF-2 release rate than KGF-P hydrogel, but slower than KGF-2 solution (Figure 4L).

### Cellular assessment of KGF-NO-MBs-P hydrogel

#### Cytotoxicity

**Figure 5.**
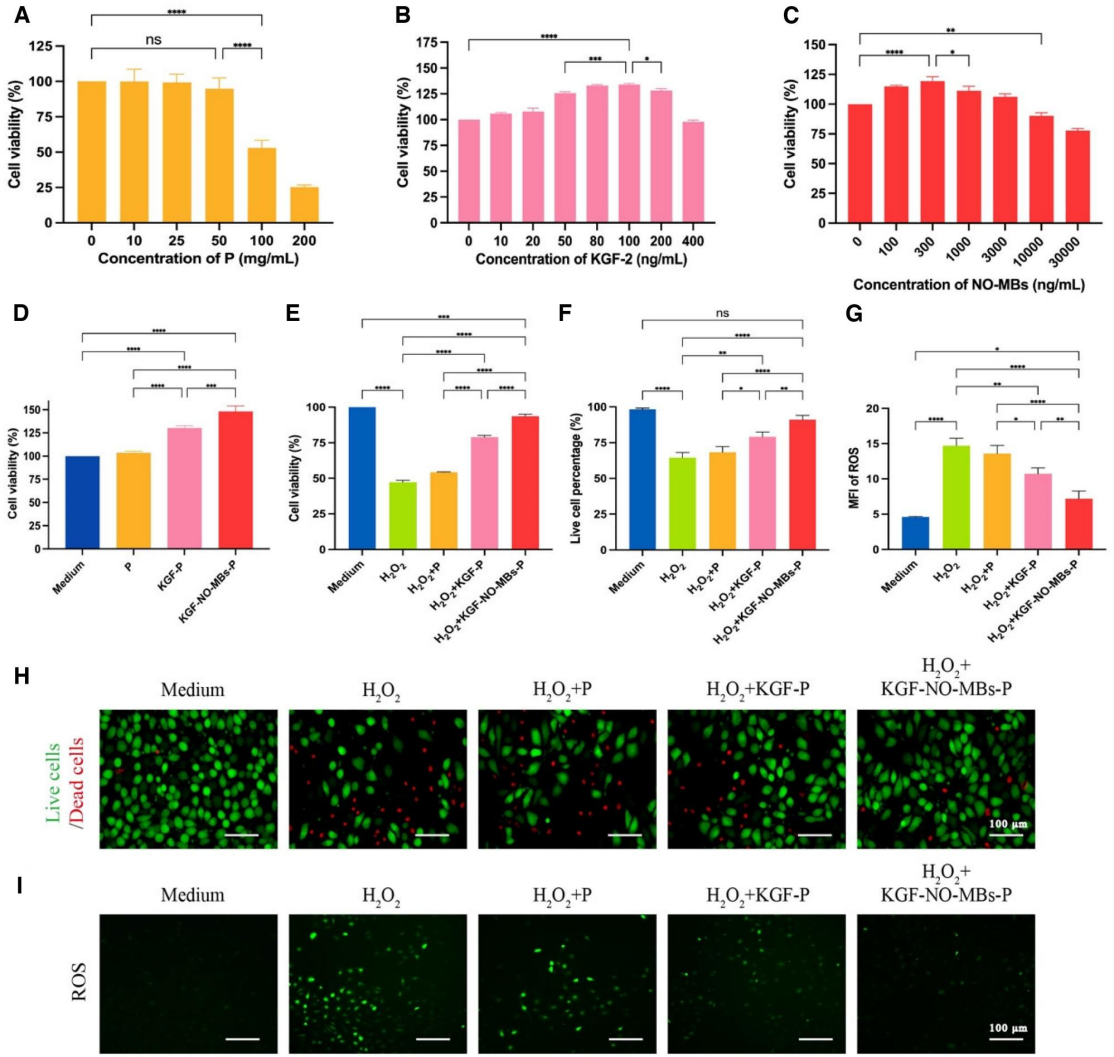
*In vitro* cytotoxicity and effect of KGF-NO-MBs-P hydrogel. (**A–C**) Cell viability of HUVECs treated with P-407, KGF-2 and NO-MBs (combined with 100 ng/mL KGF-2). (**D**) Cell viability of HUVECs treated with P hydrogel, KGF-P hydrogel and KGF-NO-MBs-P hydrogel. (**E**) Cell viability of HUVECs treated with P hydrogel, KGF-P hydrogel and KGF-NO-MBs-P hydrogel after H_2_O_2_ pretreatment. (**F** and **H**) Quantitative analysis and fluorescence images of live cells treated with P hydrogel, KGF-P hydrogel and KGF-NO-MBs-P hydrogel after H_2_O_2_ pretreatment. (**G** and **I**) Fluorescence intensity and images of ROS in HUVECs treated with P hydrogel, KGF-P hydrogel and KGF-NO-MBs-P hydrogel after H_2_O_2_ pretreatment. *P < 0.05, **P < 0.01, ***P < 0.001, ****P < 0.0001, ns, not significant.

Appropriate concentrations of P-407, KGF-2 and NO-MBs were determined by cell viability tests. Cells exposed to P-407 over 50 mg/mL had significantly decreased viability compared to Control Group, while P-407 within 50 mg/mL had no significant toxic effects on cells (Figure 5A). When concentration of KGF-2 was 10–200 ng/mL, cell viability was higher than that of Control Group. Notably, cell viability increased significantly with increasing concentration of KGF-2, and it was highest at 100 ng/mL KGF-2 (Figure 5B). Furthermore, based on the obtained KGF-2 concentration of 100 ng/mL, cytotoxicity of NO-MBs above 3000 ng/ml was detected, while NO-MBs within 3000 ng/mL had significantly increased cell viability compared to Control Group. The highest cell viability was observed at the NO-MBs concentration of 300 ng/mL (Figure 5C). Based on these, 50 mg/mL P-407, 100 ng/mL KGF-2 and 300 ng/mL NO-MBs were selected for cell tests.

#### Promotion of proliferation

Biocompatibility of the hydrogel was evaluated (Figure 5D). There was no significant difference between Medium Group and P Group, confirming that 50 mg/mL P-407 had no significant enhancement or toxic effect on cells. Incubation cells in KGF-P hydrogel or KGF-NO-MBs-P hydrogel resulted in a significantly increased cell viability, indicating proliferation effects. Besides, cell viability of KGF-NO-MBs-P Group was significantly higher than that of KGF-P Group (*P* < 0.001).

To verify proliferation and protective effects of hydrogels, cells were pretreated with H_2_O_2_ to mimic the injury state. Cell viability was significantly reduced after H_2_O_2_ pretreatment compared to Medium Group (*P* < 0.0001), and P hydrogel was inactive for therapy. After treatment with KGF-P hydrogel or KGF-NO-MBs-P hydrogel, cell viability was significantly increased. Furthermore, KGF-NO-MBs-P hydrogel had significantly better proliferation and protective effects than KGF-P hydrogel (*P* < 0.0001; Figure 5E).

In addition, live/dead cell staining was investigated (Figure 5F and H). Live cell percentage was significantly lower in H_2_O_2_ Group compared to Medium Group (*P* < 0.0001), but similar with H_2_O_2_+P Group. After KGF-P hydrogel or KGF-NO-MBs-P hydrogel treatment, cell survival was significantly improved, while KGF-NO-MBs-P hydrogel resulted in a higher live cell percentage than KGF-P hydrogel (*P* < 0.01), even similar with Medium Group.

#### Inhibition of oxidative stress

To investigate antioxidant effects, reactive oxygen species (ROS) were analyzed (Figure 5G and I). ROS expression was substantially increased after H_2_O_2_ pretreatment compared to Medium Group (*P* < 0.0001). However, this increase was not improved by P hydrogel. In contrast, ROS expression was significantly reduced by KGF-P hydrogel or KGF-NO-MBs-P hydrogel. Moreover, ROS expression was obviously lower in KGF-NO-MBs-P Group than in KGF-P Group (*P* < 0.01).

#### Promotion of tube formation and cell migration

**Figure 6.**
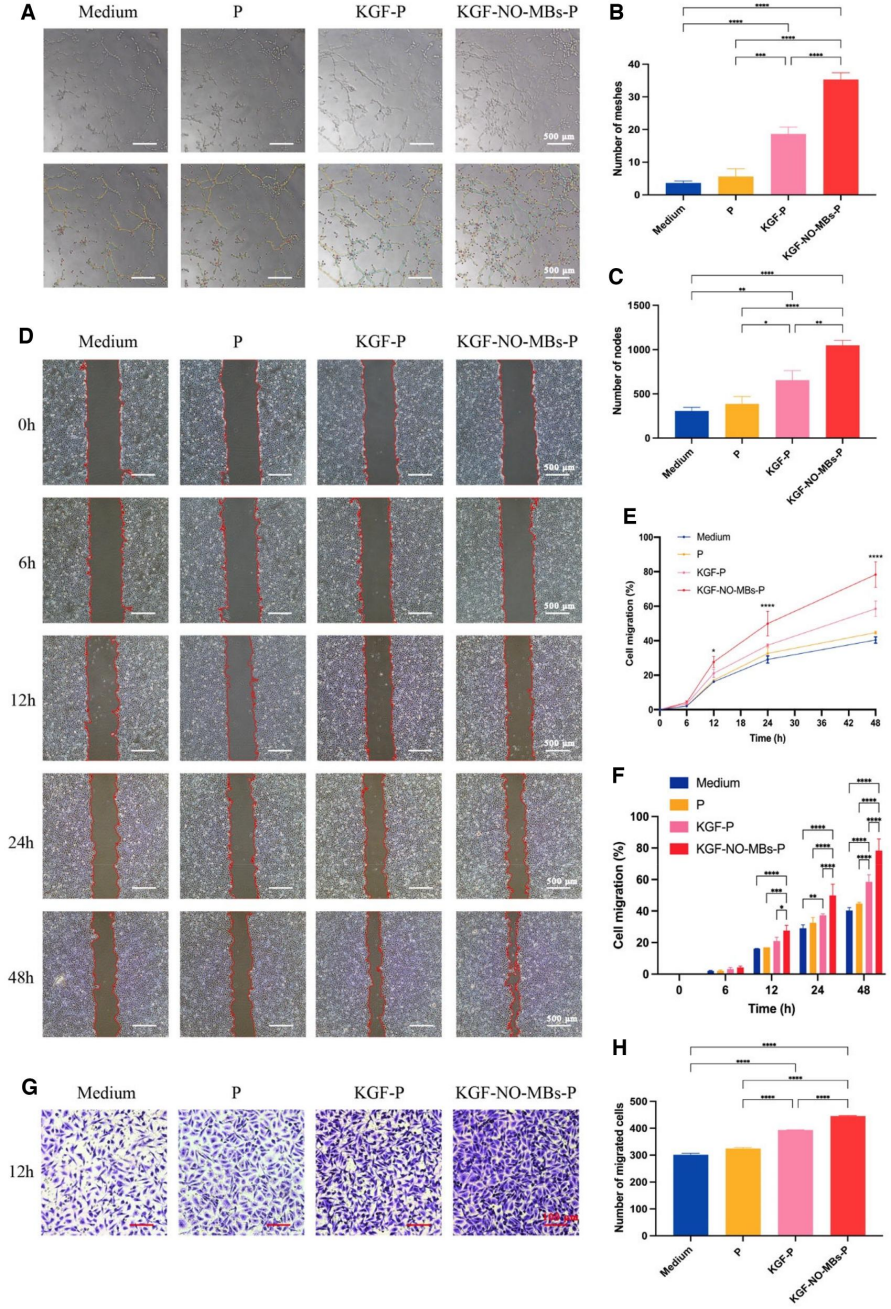
The impact of KGF-NO-MBs-P hydrogel on tube formation and cell migration. (**A**) Tube formation assay images of HUVECs treated with P hydrogel, KGF-P hydrogel and KGF-NO-MBs-P hydrogel. (**B** and **C**) Quantitative analysis of meshes and nodes. (**D**) Cell scratch assay images of HUVECs treated with P hydrogel, KGF-P hydrogel and KGF-NO-MBs-P hydrogel. (**E** and **F**) Analysis of cell migration by measuring scratch areas. (**G**) Transwell migration assay images of HUVECs treated with P hydrogel, KGF-P hydrogel and KGF-NO-MBs-P hydrogel. (**H**) Quantitative analysis of migrated cells. *P < 0.05, **P < 0.01, ***P < 0.001, ****P < 0.0001, ns, not significant.

Cellular tubule formation is shown in Figure 6A–C. P hydrogel had no significant enhancement on tube formation. However, the numbers of meshes and nodes were significantly increased in KGF-P Group and KGF-NO-MBs-P Group. Notably, KGF-NO-MBs-P hydrogel had the strongest promotion effect on tube formation and more intact tubular structures.

Cell migration was quantified by scratch healing. Cells in KGF-NO-MBs-P Group showed fastest migrating rate than other groups (Figure 6D–F). After 48 h, there was no statistical difference in scratch healing rate between P Group and Medium Group. Scratch healing outcomes in KGF-P Group and KGF-NO-MBs-P Group were significantly promoted. KGF-NO-MBs-P Group showed the higher scratch healing rate than KGF-P Group (*P* < 0.0001).

Transwell migration tests were consistent with scratch healing results (Figure 6G and H). After 12 h, cell migration numbers in P Group did not increase significantly compared to Control Group. Both KGF-P hydrogel and KGF-NO-MBs-P Group obviously promoted migration of cells. The promotion was further enhanced in KGF-NO-MBs-P group than in KGF-P Group (*P* < 0.0001).

### Evaluation of endometrial regeneration by KGF-NO-MBs-P hydrogel

#### Morphologic recovery of injured endometrium

**Figure 7.**
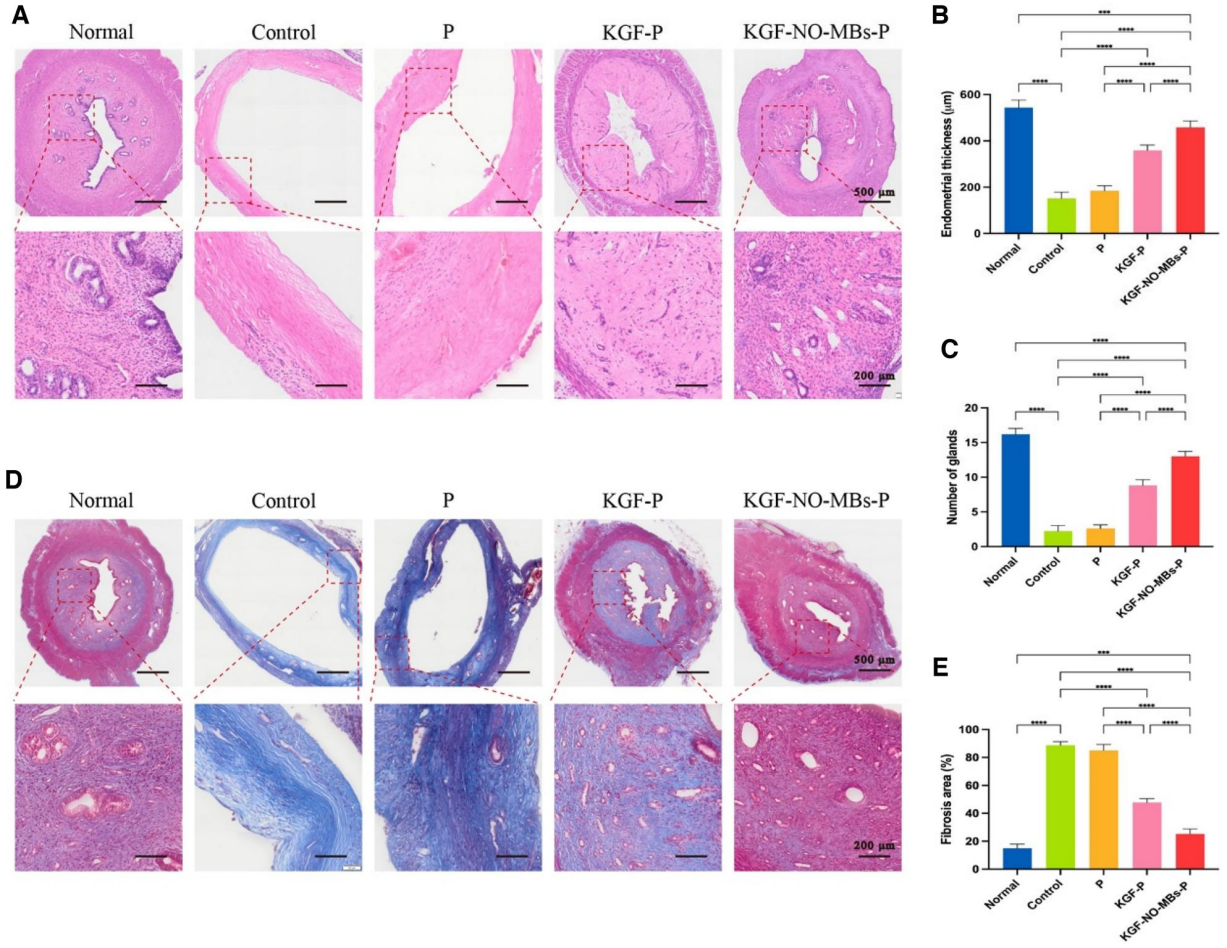
Assessment of treatment-related impact in uterine morphology and fertility. (**A**) H&E staining images of uterus in each group. (**B** and **C**) Quantitative analysis of endometrial thickness and number of glands. (**D**) Masson's staining images of uterus in each group. (**E**) Relative analysis of fibrosis areas. *P < 0.05, **P < 0.01, ***P < 0.001, ****P < 0.0001, ns, not significant.

Effects of different groups on endometrial regeneration were analyzed by H&E staining (Figure 7A–C). In Normal Group, the endometrial surface was lined with simple high columnar epithelial cells, with round or oval glands in submucosa and basal layer, as well as continuous and complete structure of myometrium. By contrast, in Control Group, there was obstruction, stenosis of uterine tissues, with destructed epithelium and glands, even the muscle fibers in myometrium were necrotic. Compared with Normal Group, the endometrium thickness and the number of glands were obviously decreased in Control Group after anhydrous ethanol-induced injury (*P* < 0.0001). In P Group, although morphology of endometrium was slightly restored due to the barrier effect of P hydrogel, these parameters were not significantly improved. However, both KGF-P hydrogel and KGF-NO-MBs-P hydrogel markedly thickened endometrium and increased the number of glands. Moreover, morphology restoration in KGF-NO-MBs-P Group was obviously superior to that in KGF-P Group (*P* < 0.0001).

Degree of endometrial fibrosis was evaluated by Masson’s staining (Figure 7D and E). There was almost no collagen deposition in endometrial stroma of Normal Group. However, compared with Normal Group, fibrosis area in Control Group was significantly increased, both in endometrial layer and myometrium (*P* < 0.0001), and P hydrogel did not significantly improve deposition of collagen fibers. In contrast, fibrotic area was significantly reduced after treatment with KGF-P hydrogel or KGF-NO-MBs-P hydrogel. The inhibition of fibrosis was markedly better in KGF-NO-MBs-P Group than in KGF-P Group (*P* < 0.0001).

#### Regeneration of angiogenesis

**Figure 8.**
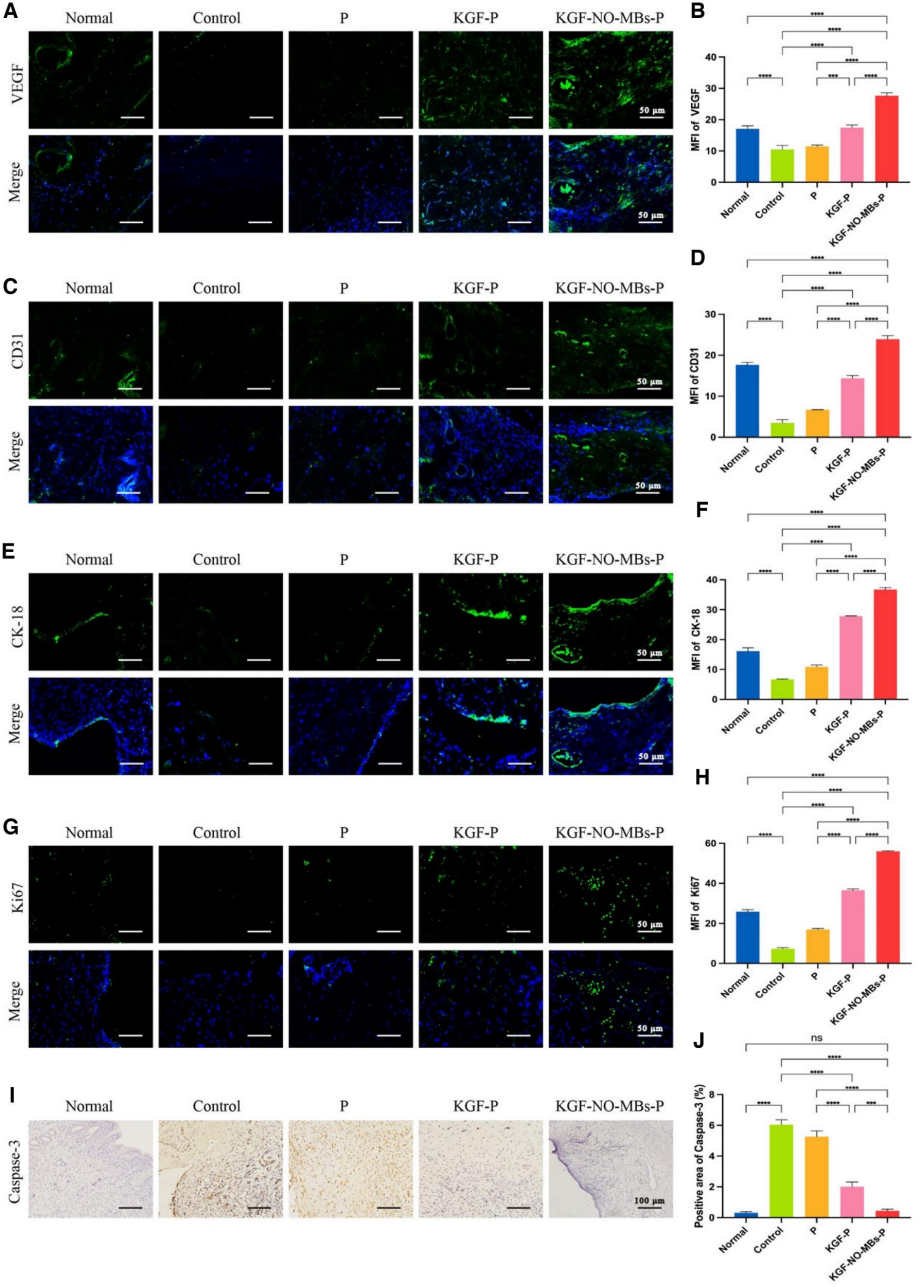
Administration of KGF-NO-MBs-P hydrogel promoted angiogenesis, epithelium proliferation and inhibited apoptosis. (**A** and **B**) Immunofluorescent staining images and mean fluorescence intensity (MFI) of VEGF in each group. (**C** and **D**) Immunofluorescent staining images and MFI of CD31 in each group. (**E** and **F**) Immunofluorescent staining images and MFI of CK-18 in each group. (**G** and **H**) Immunofluorescent staining images and MFI of Ki67 in each group. (**I** and **J**) Immunohistochemical staining images and quantitative analysis of Caspase-3 in each group. *P < 0.05, **P < 0.01, ***P < 0.001, ****P < 0.0001, ns, not significant.

Angiogenesis and vascular remodeling of endometrium were characterized by VEGF and CD31 (Figure 8A–D). The expression of VEGF and CD31 was significantly lower in Control Group compared to Normal Group, with almost no neovascularization (*P* < 0.0001). The level of CD31 was increased in P Group without obvious neovascularization, whereas the expression of VEGF was still similar with that in Control group. After treatment with KGF-P hydrogel or KGF-NO-MBs-P hydrogel, the levels of VEGF and CD31 were significantly increased, and they were higher in KGF-NO-MBs-P Group with most pronounced neoplastic micro-vessels than in KGF-P Group (*P* < 0.0001).

#### Promotion of epithelium and proliferation

The expression of CK-18 was evaluated to assess re-epithelialization of damaged endometrium (Figure 8E and F), mainly on the luminal epithelium of endometrium. Cytokeratin-positive epithelial cells were seen on endometrial surface in Normal Group. In Control Group, there was almost no CK-18 expression, which was slightly increased in P Group. The levels of CK-18 were significantly upregulated in both KGF-P Group and KGF-NO-MBs-P Group, with obvious endometrial epithelial cells. In addition, KGF-NO-MBs-P Group showed significantly higher CK-18 expression than KGF-P Group (*P* < 0.0001).

The expression of Ki67 was assessed for proliferation effects of endometrium (Figure 8G and H). Ki67 expression was obviously weaker or nearly absent in Control Group compared with Normal Group (*P* < 0.0001), and it was increased after treatment with P hydrogel. Compared with Control Group and P Group, the levels of Ki67 were significantly increased after treatment with KGF-P hydrogel or KGF-NO-MBs-P hydrogel, and it was higher in KGF-NO-MBs-P Group than in KGF-P Group (*P* < 0.0001).

#### Inhibition of apoptosis

The expression of Caspase-3 was analyzed for anti-apoptotic ability. Figure 8I and J showed that Caspase-3 levels significantly increased in Control Group compared to Normal Group (*P* < 0.0001), while there was no significant difference between P Group and Control Group. However, a significant decrease in Caspase-3 level was observed in KGF-P Group or KGF-NO-MBs-P Group compared to Control Group. Notably, in KGF-NO-MBs-P Group, Caspase-3 expression was obviously lower than in KGF-P Group (*P* < 0.001), and even comparable to Normal Group.

#### Inhibition of inflammation

**Figure 9.**
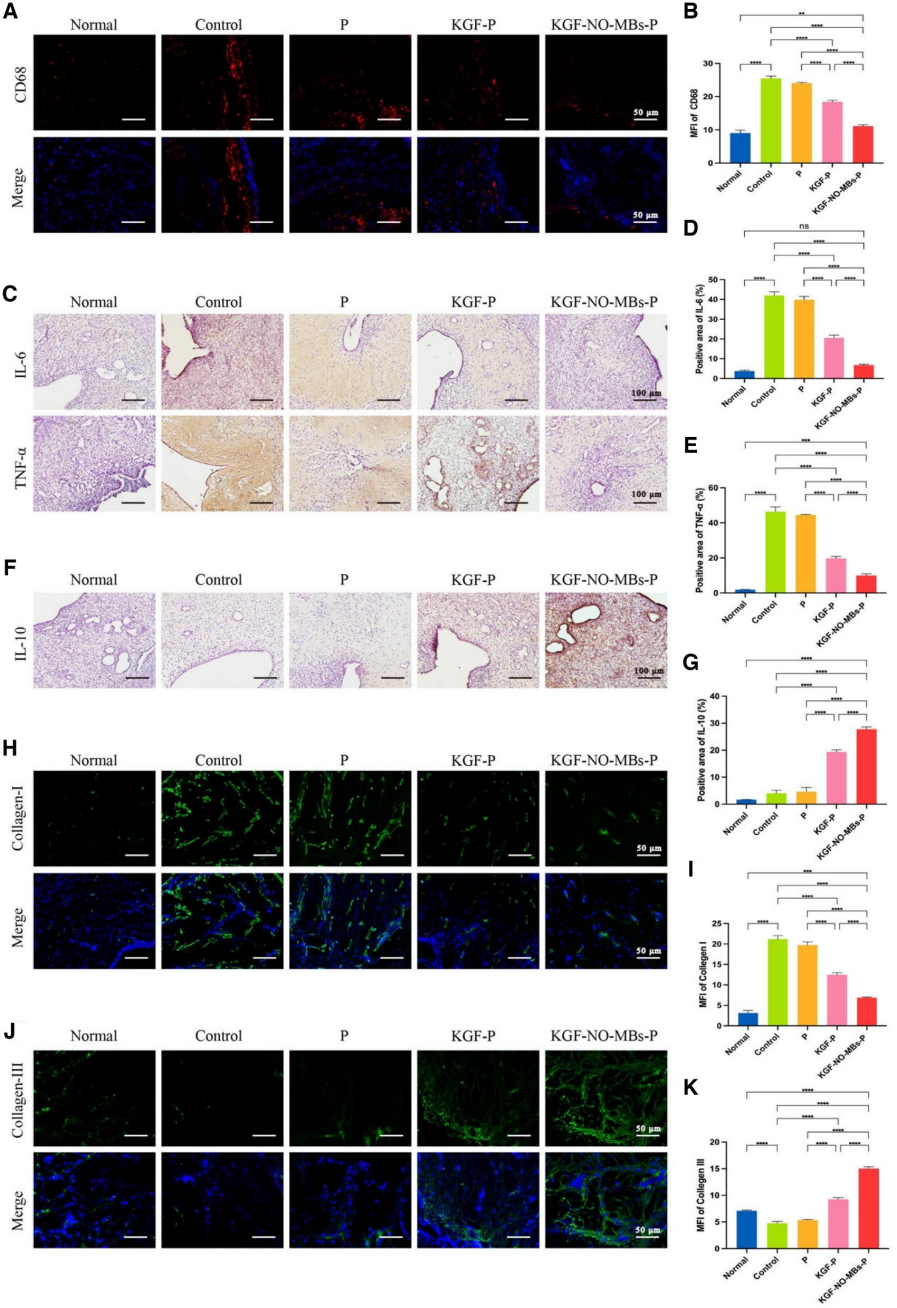
Administration of KGF-NO-MBs-P hydrogel inhibited inflammation and maintained extracellular matrix homeostasis. (**A** and **B**) Immunofluorescent staining images and MFI of CD68 in each group. (**C–E**) Immunohistochemical staining images and quantitative analysis of IL-6 and TNF-α in each group. (**F** and **G**) Immunohistochemical staining images and quantitative analysis of IL-10 in each group. (**H** and **I**) Immunofluorescent staining images and MFI of Collagen-I in each group. (**J** and **K**) Immunofluorescent staining images and MFI of Collagen-III in each group. *P < 0.05, **P < 0.01, ***P < 0.001, ****P < 0.0001, ns, not significant.

The potential of inflammation suppression effects was assessed. As shown in Figure 9A–E, abundant expression of pro-inflammatory factors IL-6, TNF-α and CD68 were observed in Control Group and P Group, with no statistical difference between two groups. Treatment with KGF-P hydrogel and KGF-NO-MBs-P hydrogel significantly reduced the expression of IL-6, TNF-α and CD68 compared to Control Group. Besides, the levels of IL-6, TNF-α and CD68 were further reduced in KGF-NO-MBs-P Group than in KGF-P Group (*P* < 0.0001). In terms of anti-inflammatory factors, Normal Group hardly expressed IL-10, while the levels of IL-10 were also weak in Control Group and P Group. As expected, IL-10 levels were significantly upregulated after treatment with KGF-P hydrogel or KGF-NO-MBs-P hydrogel, and it was even higher in KGF-NO-MBs-P Group than in KGF-P Group (*P* < 0.0001; Figure 9F and G).

#### Maintenance of extracellular matrix homeostasis

To assess collagen composition and scarless wound repair, collagen isoforms were detected (Figure 9H–K). The level of Collagen-I increased and the level of Collagen-III decreased significantly in Control Group and P Group compared to Normal Group (*P* < 0.0001). As expected, treatments with KGF-P hydrogel or KGF-NO-MBs-P hydrogel significantly decreased the level of Collagen-I but increased the level of Collagen-III compared to Control Group. Notably, KGF-NO-MBs-P Group exhibited a higher Collagen-III/Collagen-I ratio than KGF-P Group (*P* < 0.0001), suggesting a stronger effect on maintaining extracellular matrix homeostasis.

#### Fertility restoration

**Figure 10.**
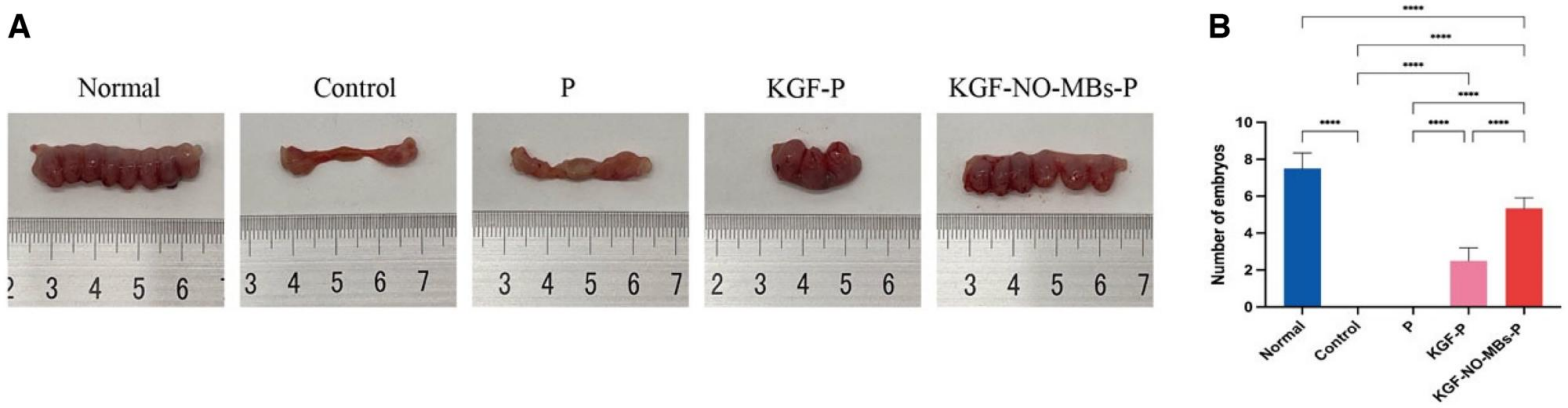
Assessment of treatment-related impact in fertility. (**A**) Images of uterus with embryo implantation in each group. (**B**) Quantitative analysis of embryo numbers. *P < 0.05, **P < 0.01, ***P < 0.001, ****P < 0.0001, ns, not significant.

Functional recovery of repaired endometrium was assessed by fertility of each group (Figure 10A and B). Pregnancy rate in Normal Group was 100% (6/6), while it was 0% (0/6) in both Control Group and P Group (*P* < 0.01). In KGF-P Group, pregnancy rate slightly increased to 33.3% (2/6). However, pregnancy rate was 50% (3/6) in KGF-NO-MBs-P Group, significantly higher than Control Group and P Group (*P* < 0.05). In addition, Normal Group had 7.5 ± 0.8 embryos, whereas Control Group and P Group both had no embryo (*P* < 0.0001). Embryos in KGF-P Group (2.5 ± 0.7) and KGF-NO-MBs-P Group (5.3 ± 0.6) were maintained until late pregnancy, significantly higher than Control Group and P Group (*P* < 0.0001). And the number of embryos in KGF-NO-MBs-P Group was obviously higher than KGF-P Group (*P* < 0.0001). These results demonstrated that KGF-NO-MBs-P hydrogel had a more potent impact on fertility enhancement.

## Discussion

The major obstacles encountered in the field of endometrial injury repair are in two main areas: angiogenesis, which has a direct impact on blood supply and nutrient access to the tissue; and re-epithelialization, which is critical to rebuilding epithelial cell layers at surface of the injury. In addition to this, excessive inflammatory response may exacerbate injury, and scar formation seriously affects repair processes. In view of the above challenges, this study innovatively adopted a two-in-one combination strategy of KGF-2 and NO, aiming to directly target angiogenesis and re-epithelialization. The combination also has ability to inhibit inflammatory responses, helping to prevent formation of scars. This study also constructed a poloxamer hydrogel drug-carrying system. This system is not only capable of stably combining two therapeutic factors, KGF-2 and NO, to achieve synchronized release but also provides a suitable microenvironment to promote penetration and retention of drugs at the site of injury.

Currently, drug delivery systems, especially *in situ* hydrogels, not only provide a physical barrier to prevent adhesion [27], but also carry therapeutic agents to repair injuries [28]. Among them, Poloxamer-407 has a controlled phase transition temperature, which is more suitable for *in vivo* drug delivery. Gelling temperature, rheology, viscosity and bio-adhesion properties are essential elements for formulation optimization and clinical application. Addition of KGF-2 decreased gelling temperature, while NO-MBs increased it in a dose-dependent manner, to adapt to abdominal temperature of rats during operation. On the contrary, apparent viscosity and adhesion were enhanced by KGF-2, but dose-dependently weakened by NO-MBs. Even so, KGF-NO-MBs-P hydrogel showed prolonged retention and restricted distribution in uterine cavity of rats. In addition, bio-adhesion was also related to rheological capacity, and KGF-NO-MBs-P hydrogel exhibited good mechanical stability and strain tolerance, which were also confirmed by other studies [29].

P hydrogel consists of hydrophilic polymers that form a polymer mesh carrying drug molecules. SEM images showed that spongy porous 3D microstructures of P hydrogel effectively loaded microbubbles without structural damage of hydrogel. Besides, it was reported that P hydrogel did not affect the size of NO-MBs (0.8 ± 0.4 μm), and half-life of NO-MBs in P hydrogel (1.21 ± 0.13 h) was longer than that in NO-MBs solution (0.81 ± 0.11 h) [30]. Similar with this study, disappearance of NO-MBs in NO-MBs-P hydrogel was slower than that in NO-MBs solution, suggesting enhancement of microbubble stability by P hydrogel. On the other hand, restricted mobility of crosslinks with porosity helps in controlled release of medications. In this study, NO was rapidly released from NO-MBs solution, but is sustainably released from P hydrogel, similar to previous studies [31]. Notably, due to affinity of P hydrogel with KGF-2, release rate of KGF-2 in KGF-P hydrogel was reported to be slow [24], as was also the case in this research. Addition of NO-MBs accelerated release rate of KGF-2, probably because of numerous microbubble pores in hydrogel matrix caused by NO release. Thus, P hydrogel is ideal for controllable delivery of KGF-2 and NO, reducing side effects of rapid drug release, administration times and doses.

Angiogenesis is a process in which endothelial cells proliferate, migrate and form ducts to generate new blood vessels, which is essential for nutritional needs of repairing tissues [32]. VEGF is an important regulator of vascular morphogenesis, which is involved in endothelial cell proliferation and mobilization, angiogenesis and vessel remodeling. CD31 is also an important vascular endothelial cell marker. We found significantly elevated levels of VEGF and CD31 with improved neovascularization in KGF-P Group and KGF-NO-MBs-P Group. Moreover, KGF-NO-MBs-P hydrogel more obviously enhanced angiogenesis results compared to KGF-P hydrogel. NO has been shown to promote recovery of blood flow in skin wounds, with underlying mechanism related to direct stimulation of VEGF expression [33]. VEGF are thought to stimulate neovascularization through NO-dependent mechanisms [34]. In addition, KGF was also discovered as a member of vascular endothelial growth factors, regulating organogenesis and angiogenesis [35]. It has been reported that KGF provokes growth of microvascular endothelial cells. Exogenous KGF was found to stimulate growth of endometrial spiral arteries [36]. Overall, compared to the use of KGF-2 alone, synergistic effect of KGF-2 and NO more effectively promotes endometrial angiogenesis.

Endometrial regeneration is associated with cell proliferation and epithelialization. Cytokeratin maintains organelle integrity and continuity through cell mitosis and differentiation, which is the marker of endometrium glandular epithelial cells [37]. CK-positive epithelial cells can be found on uterus luminal surface and in endometrial glands of normal uterus [38]. In the previous studies, there was no epithelial-like cell or weak expression of cytokeratin in injury group, while a luminal structure undercoated by epithelial cells was found in therapy group, which was positive for cytokeratin [39]. Besides, Ki67 is a marker of cell proliferation, while Caspase-3 is associated with apoptotic pathway. In this study, the levels of CK-18 and Ki67 were higher in KGF-NO-MBs-P Group than in KGF-P Group significantly, but the level of Caspase-3 was lower, although both groups restored morphology of damaged uterus compared to Control Group. KGF family is a potent repair factor for epithelial tissues [40]. Studies have shown that KGF family exhibited cytoprotective and anti-apoptotic effects by binding to FGFR2-IIIb, which was expressed by epithelial cells in tissues including endometrium [41]. KGF expression was significantly upregulated in various tissues after acute injury to facilitate recovery [42]. KGF protected cells from oxidative stress and inhibited apoptosis in injured skin [43], and also promoted proliferation of intestinal epithelial cells and attenuated inflammatory responses in rats with colitis [44]. In uterus, exogenous KGF promoted endometrial gland development in neonatal mice [45] and blocked apoptosis of basal gland cells during luteal-follicular transition [46]. In addition, appropriate low concentrations of NO can promote cell proliferation and anti-apoptotic responses, while excess NO induced cell cycle arrest, senescence and apoptosis [47]. It was found that apoptosis and oxidative stress were attenuated in hypoxic/reoxygenated cells after treatment with NO-MBs [16]. Thus, KGF-2 and NO have better synergistic effects of promoting proliferation, re-epithelialization and anti-apoptosis on endometrial cells compared to monotherapy of KGF-2.

Promotion of proliferation is associated with microenvironment restoration through inhibiting inflammation [48]. Excessive inflammation makes transition to proliferative phase difficult and is the main cause of chronic wounds [49]. Endometrial repair is similar to common trauma repair processes, facing three phases including inflammation, tissue repair and tissue remodeling [50]. Inflammation has been shown to impede recovery and induce deposition of fibrotic tissue [51]. NO is an endogenous regulator of inflammatory factors, promoting neutrophil migration and coordinating leukocyte recruitment. NO is also an antimicrobial agent with little resistance, through combination of nitrosative and oxidative mechanisms, reducing bacterial invasion at wound site [52]. It was shown that NO acted principally through immunomodulation of macrophages, presenting a marked M1-to-M2 macrophage transformation [30]. In addition, KGF-2 modulates inflammation in context of disease [53]. It has been revealed that KGF-2 could decrease inflammatory response in acute lung injury induced by oleic acid [54]. KGF-2 could reduce release of proinflammatory cytokines by regulating HMGB1-TLR4 pathway [55]. Furthermore, P hydrogel also has anti-inflammatory effect due to its isolative barrier against bacteria. In this study, compared to KGF-P Group, KGF-NO-MBs-P Group further significantly reduced the expression of pro-inflammatory cytokines and increased the expression of anti-inflammatory cytokines, showing a synergistic anti-inflammation effect of KGF-2 and NO compared to KGF-2 alone.

Abnormal repair of endometrium can lead to fibrosis, mainly including excessive accumulation of extracellular matrix followed by scar formation [56]. As a fibrillar collagen, Collagen-III is expressed in the normal endometrium and involved in blood vessel development and embryonic development, which holds great significance for endometrial regeneration [57]. Collagen-I is fibrosis-related indicator [58]. The content of Collagen-I increased after endometrial injury, while Collagen-III significantly decreased, and exogenous Collagen-III could repair the damaged endometrium [59]. Collagen-III could also remodel the endometrial immune microenvironment [60]. Studies have reported that ratio of Collagen-III/Collagen-I was highly correlated with scar formation [58]. In early stages of repair, elastic Collagen-III is expressed, while in later stages, stiffer Collagen-I is expressed. In severe cases of endometriosis, Collagen-I is dominant, while mild fibrosis retains Collagen-III, indicating the composition of collagen affects severity of fibrosis [61]. A recent study showed that Collagen-III can bind with TGF-β to attenuate signaling, showing a significant inhibitory effect on tissue fibrosis [62]. In our study, in KGF-P Group or KGF-NO-MBs-P Group, Collagen-I expression was significantly reduced while Collagen-III was abundantly expressed. Additionally, KGF-NO-MBs-P Group showed the highest Collagen-III/Collagen-I ratio, avoiding cicatrices, which was also confirmed by the most notable reduction of fibrosis areas. It was reported that the combination of KGF-2 and FGF-21 could increase the expression of Collagen-III and promote healing of burned skin wounds [63]. Besides, NO has also been shown to be effective in treatments of diabetic foot ulcer tissues by increasing ratio of Collagen-III/Collagen-I, thus, repairing ulcer wounds and reduced scar formation [30]. Totally, KGF-2 and NO synergistically promoted dynamic balance of collagen subtypes with stronger antifibrotic activity, contributing to scarless endometrium.

Restoring fertility is the primary goal of treating endometrial injury, with endometrial function evaluated by embryo implantation ability. Embryo implantation is a complex process that involves embryo quality, endometrium receptivity and coordination between embryo and endometrium development [64]. In treating endometrial injury, endometrial receptivity is crucial for assessing fertility prognosis. A clinical study confirmed that endometrial thickness can predict endometrial receptivity [65]. Uterine glands and their secretions are essential for survival and implantation of peri-implantation blastocysts, as well as for establishing uterine receptivity [66]. We found that KGF-2 and NO contributed to thicker endometrium and more glands. Besides, embryo implantation and development need a complete vascular network. Enhancing angiogenesis and intrauterine blood flow could improve endometrial receptivity [67]. We found that KGF-2 and NO could significantly increase the expression of VEGF and CD31. Upregulation of VEGF during embryo implantation enhances endometrial receptivity, regulates villous angiogenesis and promotes embryo adhesion in early pregnancy [68]. Furthermore, Collagen-III is present at the maternal-fetal interface during blastocyst implantation, supporting trophoblast and facilitating embryo implantation [69]. In this study, Collagen-III expression was significantly increased after treatment of KGF and NO. Collagen-III could bind to integrins, affecting cell proliferation, migration and adhesion [61]. Integrin αVβ3 on cell membranes in embryo and endometrium can bind to osteopontin, facilitating embryo adhesion to the endometrium. The expression of integrin αVβ3 is spatiotemporally specific, coinciding with the implantation window and serving as an indicator of endometrial receptivity [5]. In conclusion, KGF and NO could effectively regenerate endometrium, enhancing embryo implantation conditions and increase the chances of a successful pregnancy.

There are some limitations that need to be pointed out: (i) Sample sizes of this study ought to be increased, and randomized controlled trials are needed to confirm therapeutic efficacy; (ii) Signaling pathways involved in interaction of KGF-2 and NO with endometrium need to be further explored; (iii) Health and growth tests are needed for offspring after treatment with KGF-NO-MBs-P hydrogel; (iv) Due to species difference between rodents and humans, large animal models are necessary to obtain results more relevant to human applications.

## Conclusion

In this study, we demonstrated that KGF-2 and NO synergistically promoted the restoration of endometrial morphology and fertility function. Our study emphasized a localized drug delivery approach involving KGF-2 and NO-MBs incorporated into a poloxamer scaffold, developing a microenvironment-protected hydrogel. KGF-NO-MBs-P hydrogel exhibited thermo-sensitivity, stability and adhesion, enabling controlled release of NO and KGF-2. *In vitro*, KGF-2 and NO synergistically promoted cell proliferation, migration and tube formation. *In vivo* tests revealed capacity to restore endometrial thickness and glands, suppress fibrosis and improve pregnancy outcomes by inducing angiogenesis, promoting proliferation, epithelialization and extracellular matrix homeostasis while inhibiting apoptosis and inflammation through the synergistic effects of KGF-2 and NO. Overall, KGF-NO-MBs-P hydrogel presents a promising synergistic drug approach for clinical therapy of endometrial injury.
